# Domestic Violence and Coping Strategies Among Married Adults During Lockdown Due to Coronavirus Disease (COVID-19) Pandemic in India: A Cross-Sectional Study

**DOI:** 10.1017/dmp.2021.59

**Published:** 2021-03-03

**Authors:** Priyanka Sharma, Anita Khokhar

**Affiliations:** Department of Community Medicine, Vardhman Mahavir Medical College and Safdarjung Hospital, New Delhi, India

**Keywords:** COVID-19, domestic violence, lockdown, coping strategies

## Abstract

**Background:**

There has been a reported increase in cases of domestic violence during the coronavirus disease 2019 (COVID-19) pandemic; however, systematic research data are still unavailable. This study was conducted to find out domestic violence prevalence and coping strategies among married adults during lockdown due to COVID-19 in India.

**Methods:**

A cross-sectional study was conducted among married men and women in the month of April 2020. Data regarding socio-demographic profile, domestic violence, and coping strategies used during lockdown were collected thorough Google Forms. A total of 97.9% of the forms were completely filled by the respondents. A descriptive analysis was done.

**Results:**

Of 94 study participants, approximately 7.4% (*n* = 7) had faced domestic violence during lockdown. Of these 7 participants, approximately 85.7% (*n* = 6) reported increased frequency of domestic violence during lockdown. Approximately half of the victims chose to ignore it (57.1%; *n* = 4) or used yoga/meditation (42.9%; *n* = 3) to cope.

**Conclusions:**

With approximately 7.4% study participants facing domestic violence during lockdown, it is necessary to study its detailed epidemiology in pandemics so that interventions like helpline numbers, screening of patients during tele-consultation, etc., which can be delivered even during lockdown with the help of health-care and frontline workers could be devised to address this problem.

Domestic violence takes its form into physical, verbal, sexual, and economic violence.^1^ Violence, including domestic violence, against women is a public health problem across the world, and it affects one-third women.^2^ According to the Centers for Disease Control and Prevention (CDC), 1 of 4 women and 1 of 9 men faced domestic violence in United States (US) in the year 2015.^3^

The lifetime prevalence of multiple forms of domestic violence against women in India ranges from 18 to 75%, psychological abuse from 2 to 99%, physical abuse from 2 to 99%, and sexual abuse from 0 to 75%.^4^ One in 3 ever-married women has faced spousal violence at least once in their lifetime in India.^5^ Nationwide data on domestic violence against men are scarce, but a study conducted in rural Haryana showed that approximately 51.5% men had ever experienced violence by their wives or intimate partners.^6^ Physical/sexual abuse by partner can lead to injuries; mental health problems; substance use; noncommunicable diseases; poor maternal, perinatal, and reproductive health; increased chances of acquiring human immune-deficiency virus (HIV); and contracting sexually transmitted infections, such as chlamydia, syphilis, and gonorrhea. Elevated levels of acute stress and sustained stress over a prolonged duration can lead to increased susceptibility to infections.^2^

During emergencies, there is a habitual increase in violence against women, and the coronavirus disease 2019 (COVID-19) pandemic is no different.^7,8^ The pandemic has forced people to stay in home quarantines, which can aggravate the domestic violence situation and problems faced by victims as they are coerced to live in the same household with the perpetrator and, due to lockdown, are unable to go to a safe place or find a help. Disrupted social and protective networks, stress, and reduced access to services can be a catalyst in enhancing domestic violence.^8^ The domestic violence incidence could be compounded due to economic crises leading to loss of jobs and financial insecurity as victims might be dependent on the perpetrator.^9^ Although the number of reports are limited in the literature, countries across the world are seeing an increase of up to 3 times in domestic violence cases against both women and men in this COVID-19 pandemic.^7,8,10,11^

Fatke et al. reported clustering of patients presenting with psychiatric symptoms during COVID-19, including increased cases of domestic violence associated with increased drug or alcohol use in both victims and perpetrators.^12^ Another study conducted by Sediri et al. found higher scores of depression, anxiety, and stress among women who faced violence during lockdown due to the COVID-19 pandemic in Tunisia.^13^ The severity and brutality associated with domestic violence has also been on the rise in this pandemic period. Gosangi et al. reported a higher rate of physical intimate-partner violence with more severe injuries on radiology images during COVID-19 despite lesser number of patients reporting intimate-partner violence compared with past 3 y in their study conducted in United States.^14^

National Commission for Women in India has reported similar upsurge as well in number of complaints and cases of domestic violence in the country. World Health Organization (WHO) addressed this problem and urged countries to take appropriate measures to tackle this concurrent pandemic of domestic violence. However, the literature related to domestic violence during COVID-19 lockdown is limited. Therefore, the present study was conducted to find out prevalence of domestic violence and coping strategies among married men and women during lockdown in India.

## Methods

### Study Design

A cross-sectional study was conducted during the month of April 2020, after 2 wk of the nation-wide lockdown in India. The study population was composed of married men and women across the country. Inclusion criterion was currently married adult men and women.

### Study Tool and Technique

Data were collected through an online survey-based semi-structured questionnaire using Google Forms due to feasibility, which comprised of 4 sections: (1) socio-demographic profile (age, gender, religion, educational status, and occupational status of both the respondent and the spouse, socio-economic status, type of family, number of family members [numbers of male, female, and total children], substance use by the respondent or spouse, years of marriage, current pregnancy of self or spouse, extra-marital affair of self or spouse, if either of the partner had divorce in past, whether the respondent or spouse is working from home, effect of lockdown on job and income of self or the spouse, etc.); (2) prevalence and types of domestic violence faced even once in past 1 y (physical, verbal, sexual, or financial violence and the usual perpetrator of violence); (3) domestic violence faced and whether the frequency has increased during lockdown, coping strategies for domestic violence during this lockdown period (ignoring the violent incident, talking to a friend or a family member, yoga/meditation/prayers, or filing a complaint to police or a women’s helpline); and (4) who is helping them in coping (no one, friends, relatives, or neighbors). Perceived reasons for increased frequency of domestic violence during lockdown were also explored, which included uncertainty about the future, psychological or financial stress, job loss, feeling of no possible escape, etc.

Respondents were also asked about the response from police or a women’s helpline, if they had complained and various reasons for not reporting the violence, if they had not complained, including not feeling the need to report, feeling that no action will be taken against the perpetrator, anticipating improvement in the situation once lockdown is lifted, fear due to constant presence of the perpetrator in home, etc.

We also enquired if the victims had identified any safe place for escape in case the violence happens or escalates during lockdown. Socio-economic status was calculated by modified B.G. Prasad scale, which considers monthly per capita income, updated for the year 2019.^15^ The questionnaire was developed by researchers themselves after a thorough literature search. It was then pretested on a sample of 10 married adult men and women and modified based on suggestions received. The data from pretesting were not included in the final analysis. The questionnaire was then circulated through social media platforms, such as Whatsapp and emails. Convenience sampling technique was used. The investigators forwarded the Google Form link to their contacts and associates, and they were asked to forward it to their contacts further. Single response from each participant was allowed. The data were collected over a period of 1 wk from April 8, to April 14, 2020, after 14 d of nation-wide lockdown in India.

### Measures of Domestic Violence

Domestic violence in this study was defined as any act by a partner or family member residing in a joint family that harms or injures or endangers the safety and well-being of the victim as defined under the protection of women from domestic violence act, 2005.^1,4^

The forms of abuse included in domestic violence are physical, verbal, sexual, and financial abuse. For the purpose of this study, physical abuse included shoving, slapping, kicking, choking, or any other form of physical violence. Verbal abuse included yelling, name calling, abusive language, blaming, shaming, isolation, intimidation, or any other form of verbal violence. Sexual abuse included sexual assault, harassment, not allowed to use contraception, unwelcome touch, or any other form of sexual violence. Financial abuse included restriction on use/acquisition/maintenance of financial resources, stealing of money, prevented from working, or any other form of financial abuse. The perpetrators of the domestic violence could be the partner or other family members.

### Statistical Analysis

Data, entered in Foogle Forms, were converted into MS Excel spreadsheet and downloaded. It was then cleaned for errors and missing values. Statistical Packages for the Social Sciences version 17 (SPSS Inc., Chicago IL) was used for data analysis. Descriptive analysis was done using proportions, means, and standard deviation. Fischer exact test was applied to test associations between different variables and domestic violence during lockdown. A *P* value of <0.05 was considered significant.

### Ethical Statement

The study was conducted within the boundaries of Helsinki declaration. Confidentiality and privacy of data were ensured. The participants were given contact details of 1 of the authors in case they had any query.

## Results

Responses from 96 participants were received, of which 2 forms were incomplete, so they were excluded from the analysis. Therefore, final data analysis was performed for a total of 94 married adults. Mean age of the participants was 40 y (standard deviation, 10.3 y; range, 24-73 y).

More than half of the study participants were females (58.5%; *n* = 55), and the rest were males (41.5%; *n* = 39). None of the participants was from lower middle or lower socio-economic class according to B.G. Prasad scale updated for the year 2019.^15^ Almost three-fourths of the participants were residing in a nuclear family (72.3%; *n* = 68) and had up to 4 members in their family (75.5%; *n* = 71). Sixty-four study participants (68.1%) were married for 5 or more years. The majority had 1 or more children (78.7%; *n* = 74). Approximately 3.2% (*n* = 3) study participants or their spouse were pregnant at time of study. Only 2 participants or their spouse (2.1%) had divorce in past. Most of the study participants or their spouse (90.4%; *n* = 85) had not had an extra-marital affair. Less than one-fourth of the study participants reported use of alcohol (24.5%; *n* = 23) or tobacco (14.9%; *n* = 14) by themselves or their spouse. A total of 15 (16.0%) study participants or their spouse faced loss of job/income during lockdown, and 41.5% (*n* = 39) participants or their spouse were working from home (Table 1).

**Table 1.** Socio-demographic profile of study participants (N = 94)

Variable	Frequency	Percentage
Age (in completed years)		
20-29	12	12.8
30-39	41	43.6
40-49	24	25.5
50-59	9	9.6
≥60	8	8.5
Gender		
Male	39	41.5
Female	55	58.5
Religion		
Hinduism	82	87.2
Islam	6	6.4
Others	6	6.4
Educational status		
Up to senior secondary school	1	1.1
Graduate or postgraduate	55	58.5
Other professional degree	38	40.4
Educational status of spouse		
Up to senior secondary school	2	2.1
Graduate or postgraduate	64	68.1
Other professional degree	28	29.8
Occupation		
Government service	52	55.3
Private service	25	26.6
Housewife	7	7.4
Others	9	9.6
Unemployed	1	1.1
Occupation of spouse		
Government service	28	29.8
Private service	27	28.7
Housewife	17	18.1
Business	16	17.0
Others	6	6.4
Socio-economic status^a^		
Upper class (Rs. 7008 & above)	88	93.6
Upper middle class (Rs. 3504-7007)	4	4.3
Middle class (Rs. 2102-3503)	2	2.1
Lower middle class (Rs. 1051-2101)	0	0.0
Lower (below Rs. 1050)	0	0.0
Type of family		
Joint	26	27.7
Nuclear	68	72.3
Duration of current marriage (in years)		
<5	30	31.9
≥5	64	68.1
No. of family members		
Up to 4	71	75.5
5-9	18	19.1
>9	5	5.3
No. of children		
0	20	21.3
1	32	34.0
2	37	39.4
≥3	5	5.4
No. of male children		
0	38	40.4
1	49	52.1
≥2	7	7.4
No. of female children		
0	41	43.6
1	43	45.7
≥2	10	10.6
You/your spouse currently pregnant		
Yes	3	3.2
No	91	96.8
You/your spouse had divorce in past		
Yes	2	2.1
No	92	97.9
You/your spouse have an extra-marital affair		
Yes	3	3.2
No	85	90.4
May be	6	6.4
Use of alcohol by spouse and/or yourself		
Yes	23	24.5
No	71	75.5
Use of tobacco by spouse and/or yourself		
Yes	14	14.9
No	80	85.1
You/your spouse working from home during lockdown		
Yes	39	41.5
No	55	58.5
You/your spouse faced loss of job/income during lockdown		
Yes	15	16.0
No	79	84.0

a Per modified B. G. Prasad for the year 2019.^15^

Of 94 study participants, approximately 8.5% (*n* = 8) faced domestic violence in the past 1 y, and among them, the most prevalent form was verbal abuse faced by 62.5% (*n* = 5) participants while 2 participants reported facing multiple forms of violence. Approximately 7.4% (*n* = 7) of study participants faced domestic violence during lockdown. Of these 7 participants, the majority (85.7%; *n* = 6) reported an increase in frequency of violence during lockdown period. The most common type of violence that was reported to be increased during lockdown was verbal violence (57.1%; *n* = 4). The most common reason cited by participants for increase in violence during lockdown was uncertainty about the future (71.4%; *n* = 5). Other reasons cited were psychological stress, financial stress, loss of job, feeling that there is no means of escape due to lockdown, not being able to meet friends and family members, increased household responsibilities, social distancing, and spending more time together.

Of the 7 participants who faced domestic violence during lockdown, approximately half chose to ignore (57.1%; *n* = 4) and others used coping strategies, including yoga, meditation, prayers (42.9%; *n* = 3), and/or talking to a friend (42.9%; *n* = 3) and/or talking to a family member (28.6%; *n* = 2). When asked about who is helping them in coping with domestic violence, more than half of the victims (57.1%; *n* = 4) responded that they are doing it on their own. Only 1 participant had complained to police and a women’s helpline and action was taken against the perpetrator by the concerned authority (Table 2). Other participants who had faced domestic violence during lockdown but did not report it to any agency (*n* = 6) either did not feel the need to report (83.3%; *n* = 5), believed that no action will be taken due to lockdown (33.3%; *n* = 2), anticipated improvement in situation once lockdown is lifted as there will be less interaction with the spouse (33.3%; *n* = 2), were afraid due to constant presence of the perpetrator at home (16.7%; *n* = 1), were dependent on the perpetrator (16.7%; *n* = 1), or thought that domestic violence complaints from men will not be entertained (16.7%; *n* = 1) (Table 2). Most of the victims of domestic violence (75.0%; *n* = 6) had not identified any safe place to escape in case violence escalates or happens during lockdown.

**Table 2.** Prevalence of domestic violence and coping strategies among study participants

Variable	Frequency	Percentage
Faced domestic violence in past 1 y		
Yes	8	8.5
No	86	91.5
Type of domestic violence faced in past 1 y^a^ (n = 8)		
Physical	3	37.5
Verbal	5	62.5
Sexual	2	25.0
Financial	2	25.0
Usual perpetrator of domestic violence (n = 8)		
Spouse	6	75.0
Mother-in-law	2	25.0
Faced domestic violence during lockdown		
Yes	7	7.4
No	87	92.6
Increased frequency of domestic violence during lockdown (n = 7)		
Yes	6	85.7
No	1	14.3
Type of violence increased^a^ (n = 7)		
Physical	2	28.6
Verbal	4	57.1
Sexual	1	14.3
Financial	2	28.6
Possible reasons for increase in frequency^a^ (n = 7)		
Uncertainty about future	5	71.4
Psychological stress	4	57.1
Financial stress	3	42.9
Job loss	3	42.9
Feeling that there is no means of escape due to lockdown	3	42.9
Not able to meet friends, family and neighbors who are the usual stress busters or counsellors for domestic issues	3	42.9
Increased household responsibilities like doing daily chores	3	42.9
Social distancing	2	28.6
Essential duty work along with looking after a child is very problematic.	1	14.3
Spending more time together	1	14.3
Non-availability of alcohol/tobacco or other drugs	1	14.3
Increase work load at workplace	1	14.3
Coping strategies for domestic violence during lockdown^a^ (n = 7)		
Ignore	4	57.1
Yoga/meditation/prayers	3	42.9
Talk to a friend	3	42.9
Talk to a family member	2	28.6
Doing nothing	1	14.3
Complaint to police	1	14.3
Complaint to a women’s helpline	1	14.3
Who is helping in coping		
No one/on my own	4	57.1
Nearby friends	3	42.9
Relatives	3	42.9
Neighbors	2	28.6
Response received after complaint to police/women’s helpline (n = 1)		
Action taken against perpetrator	1	100.0
Why didn’t complain^a^ (n = 6)		
Did not feel the need to report	5	83.3
Feel that no action will be taken due to lockdown	2	33.3
Situation may improve after the lock down is over as there will be less interaction with the spouse	2	33.3
Fear due to constant presence of perpetrator at home	1	16.7
Financial or other dependence on perpetrator	1	16.7
Male complaints are not entertained	1	16.7
Identified any safe place if the violence escalates/happens during lockdown (n = 8)		
Yes	2	25.0
No	6	75.0

a Multiple responses present.

A significantly lower proportion of study participants who held either graduate or postgraduate degrees faced domestic violence during lockdown compared with those who were educated up to senior secondary school (*P* = 0.018). Similarly, a significantly higher proportion of study participants whose spouses were educated up to senior secondary school faced domestic violence during lockdown compared with those whose spouses held graduate or postgraduate degrees (*P* = 0.017). A higher percentage of study participants who had 2 or more male children faced domestic violence during lockdown compared with those who had no children or a single male child, and this difference was statistically significant (*P* = 0.041). Current pregnancy of self or of the spouse was found to be associated with higher prevalence of domestic violence during lockdown (*P* = 0.014). A significantly higher proportion of the study participants who themselves or whose spouses had a history of divorce faced domestic violence during lockdown compared with those who did not have divorce in past (*P* = 0. 005). Extra-marital affair of the study participants or their spouses was found to be significantly associated with domestic violence during lockdown (*P* = 0.001). Loss of job or income by either of the partner during lockdown was also significantly associated with domestic violence during lockdown (*P* = 0.011). Other socio-demographic factors were not found to be associated with domestic violence during lockdown (Table 3).

**Table 3.** Factors associated with domestic violence during lockdown (N = 94)

Variable	Domestic violence faced during lockdown		Total	*P*-Value
	Yes n (%)	No n (%)	n (%)	
Age (in completed years)				
20-29	1 (8.3)	11 (91.7)	12 (100.0)	1.000
30-39	4 (9.8)	37 (90.2)	41 (100.0)	
40-49	2 (8.3)	22 (91.7)	24 (100.0)	
50-59	0 (0.0)	9 (100.0)	9 (100.0)	
≥60	0 (0.0)	8 (100.0)	8 (100.0)	
Gender				
Male	3 (7.7)	36 (92.3)	39 (100.0)	1.000
Female	4 (7.3)	51 (92.7)	55 (100.0)	
Religion				
Hinduism	6 (7.3)	76 (92.7)	82 (100.0)	1.000
Others	1 (8.3)	11 (91.7)	12 (100.0)	
Educational status				
Up to senior secondary school	1 (100.0)	0 (0.0)	1 (100.0)	**0.018**^a^
Other professional degree	4 (10.5)	34 (89.5)	38 (100.0)	
Graduate or postgraduate degree	2 (3.6)	53 (96.4)	55 (100.0)	
Educational status of spouse				
Up to senior secondary school	1 (50.0)	1 (50.0)	2 (100.0)	**0.017**^a^
Other Professional degree	4 (14.3)	24 (85.7)	28 (100.0)	
Graduate or postgraduate	2 (3.1)	62 (96.9)	64 (100.0)	
Socio-economic status^b^				
Upper class (Rs. 7008 & above)	5 (5.7)	83 (94.3)	88 (100.0)	0.062
Upper middle class (Rs. 3504-7007)	1 (25.0)	3 (75.0)	4 (100.0)	
Middle class (Rs. 2102-3503)	1 (50.0)	1 (50.0)	2 (100.0)	
Type of family				
Joint	2 (7.7)	24 (92.3)	26 (100.0)	1.000
Nuclear	5 (7.4)	63 (92.6)	68 (100.0)	
Duration of current marriage (in years)				
<5	2 (6.7)	28 (93.3)	30 (100.0)	1.000
≥5	5 (7.8)	59 (92.2)	64 (100.0)	
No. of family members				
Up to 4	5 (7.0)	66 (93.0)	71 (100.0)	0.424
5-8	1 (5.6)	17 (94.4)	18 (100.0)	
9 or more	1 (25.0)	4 (75.0)	5 (100.0)	
No. of children				
0	0 (0.0)	20 (100.0)	20 (100.0)	0.244
1	2 (6.3)	30 (93.7)	32 (100.0)	
2	4 (10.8)	33 (89.2)	37 (100.0)	
≥3	1 (20.0)	4 (80.0)	5 (100.0)	
No. of male children				
0	0 (0.0)	38 (100.0)	38 (100.0)	**0.041**^a^
1	6 (12.2)	43 (87.8)	49 (100.0)	
≥2	1 (14.3)	6 (85.7)	7 (100.0)	
No. of female children				
0	2 (4.9)	39 (95.1)	41 (100.0)	1.000
1	4 (9.3)	39 (90.7)	43 (100.0)	
≥2	1 (10.0)	9 (90.0)	10 (100.0)	
You/your spouse currently pregnant				
Yes	2 (66.7)	1 (33.3)	3 (100.0)	**0.014**^a^
No	5 (5.5)	86 (94.5)	91 (100.0)	
You/your spouse had divorce in past				
Yes	2 (100.0)	0 (0.0)	2 (100.0)	**0.005**^a^
No	5 (5.4)	87 (84.6)	92 (100.0)	
You/your spouse have an extra marital affair				
Yes	2 (66.7)	1 (33.3)	3 (100.0)	**0.001**^a^
No	3 (3.5)	82 (96.5)	85 (100.0)	
May be	2 (33.3)	4 (66.7)	6 (100.0)	
Use of alcohol by spouse and/or yourself				
Yes	2 (8.7)	21 (91.3)	23 (100.0)	1.000
No	5 (7.0)	66 (93.0)	71 (100.0)	
Use of tobacco by spouse and/or yourself				
Yes	3 (21.4)	11 (78.6)	14 (100.0)	0.065
No	4 (5.0)	76 (95.0)	80 (100.0)	
You/your spouse working from home during lockdown				
Yes	3 (7.7)	36 (92.3)	39 (100.0)	1.000
No	4 (7.3)	51 (92.7)	55 (100.0)	
You/your spouse faced loss of job/income during lockdown				
Yes	4 (26.7)	11 (73.3)	15 (100.0)	**0.011**^a^
No	3 (3.8)	76 (96.2)	79 (100.0)	

a Significant association.

b According to modified B.G. Prasad scale for the year 2019.^15^

## Discussion

The present study attempted to find out the prevalence of domestic violence among married adult men and women during lockdown period due to COVID-19 and how are they coping with it. There were reports of increased complaints of domestic violence from various parts of world, including India; however, systematic research is limited. We tried to fill this gap through the present study, which can help in formulation of specific public health preventive measures to curb these violence incidents during times of pandemics. These preventive measures should be implemented in times of epidemics, pandemics, and other emergency situations when there is a possibility of implementation of movement restrictions.

Such situations can be predicted in advance based on data available from other countries and regions. A mechanism similar to integrated disease surveillance program (IDSP) in India, which monitors trends of epidemic prone diseases across the country and detects and responds to outbreaks in early rising phase^16^ can be developed for reporting of incidents of domestic violence which can provide early trigger for expected increase in near future. Scanning media reports, data sharing from crime records organizations such as National Crime Records Bureau (NCRB) in India and input from organizations working in alleviation of gender-based violence can help predict trends in domestic violence incidents in unprecedented times. Based on these reports, appropriate preventive measures can then be designed and implemented. First, health-care workers need to be sensitized about the possibility of increased domestic violence during lockdown. They should screen patients coming to health-care facilities or during tele-consultation routinely for any signs of abuse. This opportunity can also be used to disseminate helpline numbers to the patients. Special signs or symbolic language can be used to indicate abuse or domestic violence as it might be difficult for the victims to report such incidents in presence of perpetrator. Peripheral health-care workers, for example accredited social health activists (ASHAs) in India, can follow up on their beneficiaries as they live in the same community. Second, dedicated helpline numbers, email addresses, Whatsapp numbers can be started by the government and nongovernmental organizations where victims can report violence and seek help, if needed, and these can be communicated through radio, television, Internet, and other media sources.

As many of the victims in the present study talked to a friend or a relative about violence, community awareness is a necessity so that friends or relatives will come forward and report such incidents. They should be protected under provisions similar to the Good Samaritan law for road accidents.^17^ Third, people who had reported such incidents in past during normal times, should be followed up telephonically or in-person to check if they are facing violence during lockdown. Political commitment is another component that should be addressed. Leaders should mention the preventive strategies for this shadow pandemic of domestic violence during their media briefings and speeches.

Approximately 7.4% (*n* = 7) of study participants in the present study had faced domestic violence during this lockdown period and 8.5% (*n* = 8) faced it in past 1 y. The most prevalent form of domestic violence faced among the victims in past 1 y was verbal (62.5%; *n* = 5), followed by physical (37.5%; *n* = 3), sexual (25%; *n* = 2), and financial abuse (25%; *n* = 2), and multiple forms of violence by 2 participants (25%). This was similar to the findings of a systematic review conducted by Kalokhe et al. in which the prevalence of multiple forms of domestic violence among Indian women in past 1 y ranged from 4 to 56%, and the highest prevalence was of psychological abuse including verbal abuse, which ranged from 11 to 48%.^4^ This prevalence was lower compared with National Family Health Survey-4 data and other studies conducted across the country.^5,18-20^ The disparity in findings could be because these studies included only women participants, who were ever married, in the age group of 15-49 y and most of them have reported violence only by spouse and not by other family members. While the present study included both men and women over a wide age range and studied violence by spouse as well as other family members. Additionally, over-representation of upper and middle class literate study participants in the present study could have led to lower prevalence as it has been identified as a protective factor against domestic violence in previously conducted studies.^18-20^

The negative repercussions of domestic violence on health are innumerable including poor reproductive, sexual, physical, and mental health and adverse child health outcomes.^21^ There might be an upsurge in these conditions during disasters and pandemics owing to increases in incidents and severity of domestic violence, which was evident from the present study also where 85.7% (*n* = 6) victims reported an increase in frequency of domestic violence during lockdown period. But imposed restrictions, overburdened health staff, scaled back essential support services can lead to reduced access to help by victims during pandemics.^22^

The common coping strategies used by the victims of domestic violence during lockdown period as found in the present study were ignoring the incident, talking to a friend or family member, and doing yoga or meditation. Only 1 participant reported the domestic violence incident to police and a women’s helpline asking for help. This finding was similar to a report by United Nations according to which less than 40% of women victims of violence seek any sort of help and mostly seek help from family or friends. This report also highlighted the fact that less than 10% of the women victims who request help, ask for help from police.^23^ Similarly, there were reports of a decrease in calls to domestic violence by approximately 55% during the COVID-19 pandemic from countries like Italy and France.^23^ This is worrisome as victims with no access to help and other resources might face increased severity and frequency of violence, which could further deteriorate their health.

The most common reason cited for not complaining in the present study were not feeling the need to report or feeling that no action will be taken against the perpetrator due to lockdown. This points toward the loss of faith in the legal system and acceptance of violence by victims, which can make them more vulnerable to the domestic violence and abuse. Constant fear of the presence of the perpetrator at home was also reported to be the reason for not complaining by the victims in the present study.

Only 25% (*n* = 2) of victims who had faced domestic violence in past 1 y had identified a safe place to go in case the violence happens or escalates during lockdown. The possible reasons for this lower proportion could be not having access to finances, dependence on the perpetrator, no place to go or restrictions imposed due to lockdown itself.

There was no significant difference found in domestic violence among males and females. Approximately 7.7% of male study participants had faced domestic violence during the lockdown period. Similar prevalence of domestic violence against men in the past 1 y (10.5%) was found in a study by Malik and Nadda.^6^ Domestic violence studies have mostly been negligent toward males as victims. With changing cultural and social values, it is required to study gender associations with domestic violence in India.

A significantly higher percentage of victims who were pregnant at the time of study had faced violence compared with those who were not. Extra-marital affair of self or spouse was found to be significantly associated with domestic violence during the lockdown period. Low educational status of victims as well as spouse was also found to be significantly associated with domestic violence during lockdown. This was similar to the findings of other studies where lower educational status was a risk factor for violence.^6,20^ Loss of job/income was a significant factor for domestic violence during lockdown. This finding was reiterated in the reasons perceived by victims for increase in frequency of domestic violence during lockdown, which included job loss and financial insecurity. This was similar to the findings of international agencies’ reports.^7,8^

### Strengths

This study is among the few studies carried out during crisis times to study domestic violence. Both males and females were included in the study, and multiple forms of domestic violence were studied. Participants from a wide age group range were invited to participate. Even with the limited sample size, the study provides important information about coping strategies adopted by victims of domestic violence during the lockdown period. The reasons for not reporting such incidents to police or a women’s helpline were also explored, which is crucial for development of preventive strategies.

### Limitations

As this was an online survey-based study, selection bias was inevitable because only those participants who had access to Internet and mobile phones or computers, and were literate could participate in the survey. Participants from the upper social class were over-represented due to the study technique. So, the results cannot be generalized to other populations. The authors acknowledge this major limitation of the study as face-to-face interviews were not possible during the time of study. The small sample size of the study is not representative of all the families who had faced the consequences of lockdown. Also, underreporting of the prevalence, given the sensitivity of the issue, and information bias cannot be ruled out. The present study findings are limited in their scope of studying the effects of coping strategies in distress management among the victims of domestic violence.

There are few public health implications of this study. Increased frequency of domestic violence during lockdown is a challenge and is not gender biased. This study highlights the importance of incorporating strategies to combat domestic violence in epidemic preparedness and response planning. There were certain high-risk groups identified in this study for domestic violence during lockdown, which needs special attention while devising preventive policies. The coping strategies get limited due to lockdown restrictions, and their impact can be profound on mental health, which needs to be studied in detail further.

## Conclusion and Recommendations

The prevalence of domestic violence among married adults during lockdown was found to be 7.4% in present study. For development of strategies and interventions to check the incidents of domestic violence, further studies are required among both the genders to explore the epidemiology of domestic violence, along with factors associated with it, and this should include situations of epidemics and other unforeseen circumstances. The devised strategies should be tailor-made for both the genders, various cultures, regions, and situations.

More than half of the victims (57.1%) chose to ignore and only 14.7% of victims complained to police or a women’s helpline and one of the reasons cited for not complaining was lack of assurance of action from legal agencies. It is, therefore, required that law enforcing agencies should use social media and other platforms to give a strong message to the community that violence incidents will be given high priority even during lockdown so that victims would feel free to seek legal help and ignorance is not used as a coping strategy.^23^ Dedicated helplines should be started, and a zero tolerance approach should be maintained.
